# Giant organ confined prostatic adenocarcinoma: a case report

**DOI:** 10.1186/1752-1947-2-28

**Published:** 2008-01-29

**Authors:** Jamin V Brahmbhatt, Louis S Liou

**Affiliations:** 1 Boston University School of Medicine, Boston, MA. 715 Albany Street, Boston, MA 02139, USA

## Abstract

**Introduction:**

Giant prostatic adenocarcinoma represents a rare and challenging treatment dilemma.

**Case presentation:**

We describe a case of an otherwise healthy 71-year-old African male who presented with a PSA of 5800 ng/ml and a prostate volume of over 1000cc. Unique aspects of this case include the size of the prostate, the apparent absence of distant metastases, and the safe usage of transabdominal biopsy of this mass.

**Conclusion:**

We present this case report and review of literature to generate further discussion amongst readers as to management options for this difficult case.

## Introduction

Giant prostatic adenocarcinoma represents a rare and challenging treatment dilemma. Previous reports [1,2] describe a few cases of this condition and their initial clinical and radiologic presentation. With increased use of PSA screening since that time, there have been no additional cases reported in the recent literature. In this case report, we describe an otherwise healthy male who presented with a PSA of 5800 ng/ml and a prostate volume of over 1000cc. Unique aspects of this case include the size of the prostate, the safe usage of transabdominal biopsy of this mass, and the apparent absence of distant metastases. The authors would like to generate further discussion amongst readers as to management options for this difficult case.

## Case Presentation

A 71-year-old African male was referred from an outside hospital for further management after initially presenting with daytime frequency and nocturia. The patient reported symptomatic relief with tamsulosin 0.4 mg once a day. However, a serum PSA obtained by the primary care physician was 5874 ng/ml.

On initial presentation, he reported minimal lower urinary tract symptoms. He complained of nocturia 2–3 times per night, which had improved with one month of tamsulosin. Review of systems was negative. His past medical history was significant for hypertension treated with Atenolol 50 mg once a day. He had no known allergies and no family history of genitourinary malignancy. On physical exam, the patient was a well-nourished male in no distress. Examination of his abdomen revealed a lower abdominal suprapubic mass. Digital rectal exam revealed a firm and markedly enlarged prostate. Laboratory values and urinalysis were normal. Repeat PSA was 5620 ng/ml.

**Figure 1:**
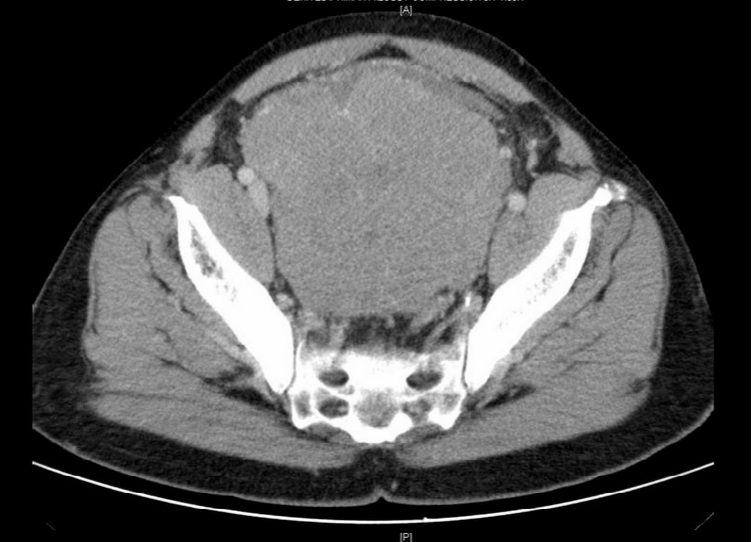
CT scan demonstrating a 12 × 13 × 10 cm (1560 cm3) mass in the pelvis. There is significant bilateral compression of the external iliac vessels and rectum.

**Figure 2:**
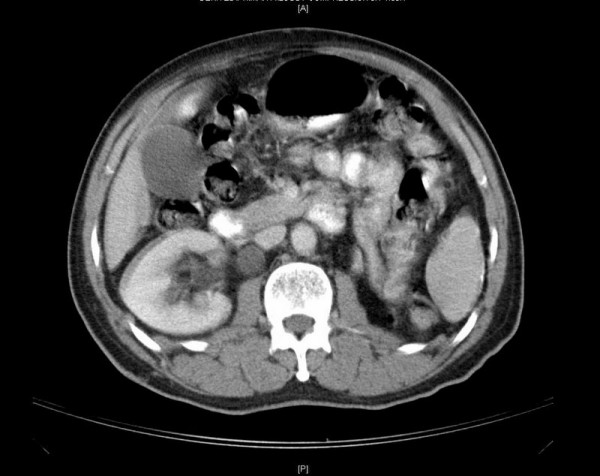
Bilateral mild to moderate hydroureteronephrosis was noted, and the left kidney appeared markedly atrophic.

A CT scan of the abdomen and pelvis demonstrated a 12 × 13 × 10 cm (1560 cm3) mass in the pelvis [Figure 1]. There was significant bilateral compression of the external iliac vessels, and compression of the rectum. Bilateral mild to moderate hydroureteronephrosis was noted, and the left kidney appeared markedly atrophic [Figure 2]. Both a nuclear medicine bone scan and plain films of the skull were negative.

**Figure 3:**
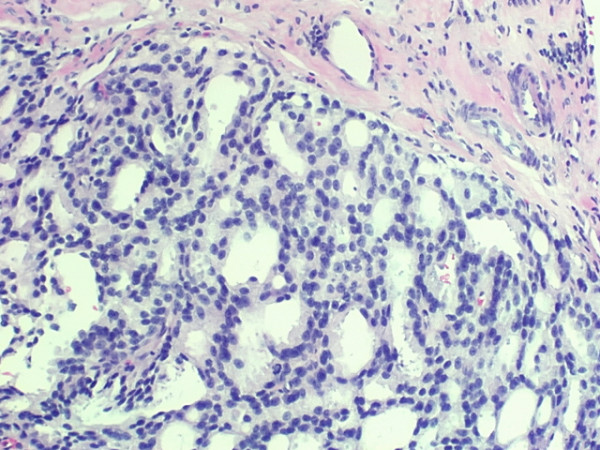
Transabdominal biopsy specimen showing Gleason 4+4 = 8 disease in greater than 70% of the prostatic tissue (hematoxylin-eosin stain).

Prostate needle biopsies with 14 cores were obtained using both a transrectal and a transabdominal approach. All biopsy specimens demonstrated Gleason 4+4 = 8 disease in greater than 70% of the prostatic tissue except for one biopsy, which showed Gleason grade 3+4 = 7 in less than 15% [Figure 3].

## Management

The patient was started on androgen ablation with bicalutamide 50 mg every day for one month and a depot leuprolide injection two weeks after the bicalutamide was started. He has then lost to follow up. No laboratory or radiological studies were repeated since the initial presentation.

## Discussion

Three issues with this case merit discussion. First, despite an elevated PSA of over 5800 ng/ml, this patient had no clinical evidence of metastatic disease on either bone scan or CT scan of the abdomen and pelvis. Others have previously reported patients with similar presentations. Stamey et al reported three such patients, all with PSA greater than 100 ng/ml, prostate size greater than 100 gm, and cancer arising from the transitional zone of the prostate [3]. All three cancers were nonpalpable on DRE, and each patient was successfully cured with surgery. It is possible that this patient represents an extreme manifestation of this phenomenon, with a massive prostate cancer and organ confined disease. Or there is also the possibility that this may be a case of giant benign prostatic hypertrophy with underlying carcinoma. This theory is unable to be verified without examination of the entire gross prostate specimen. Either way, this patient had biopsy proven cancer that required medical intervention.

Second, the usage of ultrasound guided transabdominal biopsy of this mass is rare, having previously only been described by one other institution [4]. In their patient, the suprapubic needle biopsy revealed that the mass was well differentiated prostatic adenocarcinoma. No complications were noted. In both cases, transabdominal biospy of large pelvic masses represented a safe alternative to transrectal biopsy when the transrectal approach is poorly tolerated. Furthermore, the advantage of the transabdominal approach is that anterior prostate, which is not accessible using the standard approach, can now be biopsied.

Third, this patient's treatment options appear to include only hormonal ablation and eventual chemotherapy. Similar cases in the literature were treated with hormonal ablation, but all of these patients had signs of metastatic disease at time of presentation. Recently however, Masue et al [5] described a case giant prostate carcinoma treated effectively with endocrine therapy. This patient, with no evidence of metastases, could theoretically be a candidate for neoadjuvant hormonal ablation followed by radical prostatectomy or pelvic XRT.

Hormonal ablation has been used in men with very large prostates to reduce the size for easier removal. This method has shown no demonstrable benefit in 5-year outcomes for patients undergoing radical prostatectomy [6]. Conversely, Meyer et al found a longer disease free survival when neoadjuvant hormonal ablation is used for greater than 3 months prior to surgery [7]. In another study, 4 months of neoadjuvant therapy prior to radical prostatectomy in T3 disease found pathologic downstaging to a lower stage (T2c or lower) in 48% of patients. If responsive to androgen ablation, our patient may be a candidate for surgery in the future.

The mechanical difficulties and risks of surgery along with the indefinite survival benefit make the case for prostatectomy difficult in our patient. If surgery is attempted, the large size of the prostate is likely to have distorted periprostatic anatomy, leading to poor isolation of the superficial dorsal vein, unachievable nerve sparing, and probable poor bladder neck preservation. Post-operatively the patient has high risk of incontinence, impotence, and other acute surgical morbidities. Even though surgery is the best viable option for clinically localized prostate cancer, in the case of large volume adenocarcinoma the mechanical risks may well outweigh the benefits of the procedure.

Recently, the use of radiation and androgen ablation was shown to have a significant benefit in men with clinically localized prostate cancer in high-risk groups [8]. However, no study has compared efficacy of radiation with varying volumes of prostate. It can be inferred that the large field size and high Gleason grades in large prostatic adenocarcinomas may have a low local failure rate. However, these same variables may require higher doses of radiation and lead to high levels of regional toxicity.

Cryotherapy is a promising alternative in our patient for the reduction of large prostatic neoplasms. Indications for cryoablation in our patient include localized cancer with relative contraindications to radical prostatectomy [9]. However, cryosurgery is not currently recommended in patients like ours with a prostate volume of >40 mL because the large glands may prevent adequate freezing of the prostate. Prepelica et al [10] recently found a durable PSA biochemical disease-free survival in 83.3% of patients and concluded that cryoablation is a feasible treatment option in patients with organ-confined prostate carcinoma with high-risk features (PSA ≥ 10 ng/mL, or a Gleason sum score ≥8, or both). The study, however, did not state the size of the treated prostate or the stage of disease, making it difficult to generalize the results for this case. Nonetheless, in patients with large prostatic adenocarcinoma, cryosurgery offers patients another viable treatment option.

## Conclusion

In conclusion, giant prostatic adenocarcinoma is a rare condition, but it poses many treatment dilemmas to the Urologist. As with all treatment decisions, tumor class, life expectancy, disease-free survival, treatment associated morbidity, patient preference and physician expertise must be taken into account.

## Competing interests

The author(s) declare that they have no competing interests.

## Authors' contributions

LL managed the patients care and drafted the patient presentation portion of the manuscript. JB researched and drafted the manuscript. All authors read and approved the final manuscript.

## Consent

Patient consent was received for publication of the manuscript.
